# Multi-omics analysis of the development and fracture resistance for maize internode

**DOI:** 10.1038/s41598-019-44690-6

**Published:** 2019-06-03

**Authors:** Xiaqing Wang, Ruyang Zhang, Zi Shi, Ying Zhang, Xuan Sun, Yulong Ji, Yanxin Zhao, Jidong Wang, Yunxia Zhang, Jinfeng Xing, Yuandong Wang, Ronghuan Wang, Wei Song, Jiuran Zhao

**Affiliations:** 1 Beijing Key Laboratory of Maize DNA Fingerprinting and Molecular Breeding, Maize Research Center, Beijing Academy of Agriculture and Forestry Sciences, Shuguang Huayuan Middle Road, Haidian District, No. 9, Beijing, 100097 China; 2 Beijing Key Lab of Digital Plant, Beijing Research Center for Information Technology in Agriculture, Beijing Academy of Agriculture and Forestry Sciences, Shuguang Huayuan Middle Road, Haidian District, No. 11, Beijing, 100097 China

**Keywords:** Biotic, Plant morphogenesis

## Abstract

The maize stalk is an important mechanical supporting tissue. The stalk fracture resistance is closely related to lodging resistance, and thus the yield. In this study, we showed that the basal zone (BZ) was more fragile than the middle zone (MZ) of the stalk internode before tasseling. In order to clarify the relationship between the different zones and fragile resistance between the internodes, we systematically analyzed the phenotypic, metabolomic and transcriptomic differences. The results indicated that the BZ zone had lower stalk strength, which corresponded to the results of less lignin, cellulose and hemicellulose than that of the MZ. The 27 highly enriched metabolites and 4430 highly expressed genes in the BZ mainly participated in pentose phosphate, and in ribosome and sterol synthesis pathways, respectively. In addition, the BZ had higher vascular bundles density but smaller size compared with the MZ. By contrast, the 28 highly enriched known metabolites and 4438 highly expressed genes in the MZ were mainly involved in lignin synthesis, and secondary metabolites synthesis, respectively, especially the phenylpropanoid synthesis. The results provide a deeper understanding of the relationship between development and fracture differences in stalk, and may facilitate the improvement of field management practice to reduce lodging.

## Introduction

The stalk is an important supporting organ that carries water and nutrients, and serves as a structural and non-structural carbohydrate storage organ^1^. The development of the stalk involves many important biological processes, such as cell division, cell wall synthesis, and vascular bundle formation^2^.

The plant cell wall constitutes the skeletal structure of the stalk, and determines its mechanical strength. The cell wall is a highly organized composite structure that contains cellulose, hemicellulose, lignin and other components^3^. Among these cell wall components, cellulose is the predominant class of polysaccharides^4^. Cellulose exists as an unbranched chain containing up to 15,000 β-1,4-linked glucose residues, which are connected by strong hydrogen bonds to provide structural stability (Supplementary Fig. S1A)^4^. Cellulose synthesis is catalyzed by a complex of several different cellulose synthases (CesAs), which synthesize glucan chains using UDP-glucose as a substrate^5^. Hemicelluloses are a diverse of heterogeneous group of polysaccharides which include xyloglucans, xylans, mannans and glucomannans, and mixed linkage β-(1-3, 1-4)-glucans (Supplementary Fig. S1B)^6,7^. Hemicelluloses account for an average up to 50% of the biomass of annual and perennial plants^5^. The most important biological function of hemicelluloses is their interaction with cellulose and lignin to strengthen the cell wall^8^. Lignin is a phenolic polymer that is mainly deposited in secondary cell walls, where it is cross-linked with cellulose and hemicellulose^9^. The role of lignin include mechanical support, water transport, and defense against pathogens in vascular plants. Lignin biosynthesis starts with the phenylpropanoid pathway, which generates precursors to a diverse group of compounds including coumaryl, coniferyl and sinapyl alcohols which are linked using C–O and C–C bonds in a random order to form the extended lignin network (Supplementary Fig. S1C)^10,11^.

The stalk consists of multiple repeating phytomeric units composed of a node and an internode^12^. For example, it has been shown that the internode of *Setaria viridis* can be divided into four zones, namely the intercalary meristem, cell expansion, transition, and cell maturation zones^13^. The intercalary meristem exists at the base of the internode, where the cells are characterized by strong division. The expansion zone is close to the meristem zone, in which cell expansion and synthesis of the primary cell wall occur. The transition zone is above the elongation zone, where cell expansion slows down and the secondary cell wall begins to form. At the maturation zone, cell expansion ceases^13^. Foxtail, rice, bamboo and maize exhibit a similar developmental pattern^2,14–16^.

Stalk cell wall components are different in different zones of the internode. For example, in rice culm, Lin *et al*. (2017) found that cellulose, lignin and xylose contents gradually increased from younger tissue to older tissue by divided the elongating rice internode into eight segments^15^. In addition, a large number of proteins related to cell wall biosynthesis or remodeling were identified in the booting area of the stem, such as glycosyltransferase, acyltransferase, glycosyl hydrolase, cell wall localization proteins, protein kinase, and leucine-rich repeat type III kinase family receptors^15^. To study internode development in maize, Zhang *et al*. (2014) divided the internode into 10 parts to examine the cell wall components and the gene expression characteristics^16^. They demonstrated that the 10 parts could be classified into four zones, as described by Martin *et al*.^13^. No drastic increases in cellulose, lignin, glucuronic acid and arabinoxylan were observed from the meristem to the maturation zone^16^.

Mechanical strength is an important trait of the stem, as it determines the lodging resistance of crops. However, the relationship between the mechanical resistance and various developmental stages as well as the difference in gene expression level is still unclear. Based on years of field practice, we have noticed if maize is stressed by strong wind before tasseling, the stalk is prone to break. And the breakage position tends to be at the base rather than the middle of the internode^17^. To explore the reasons for the fracture differences, we compared the differences of the basal zone (BZ) and middle zone (MZ) using the commercial inbred line JING724 which was the female parent of the maize leading variety Jingke968^18^. Based on their mechanical strengths, vascular bundle characteristics, cell wall components, metabolites, and gene expression, we established a model for the relationship between stalk fracture resistance and internode development, which not only can propose potential solutions to improve the stalk mechanical strength, but also can facilitate the study and breeding of stalk fracture resistance maize varieties.

## Results

### Internode length and rind penetrometer strength differences of the BZ and MZ

**Figure 1:**
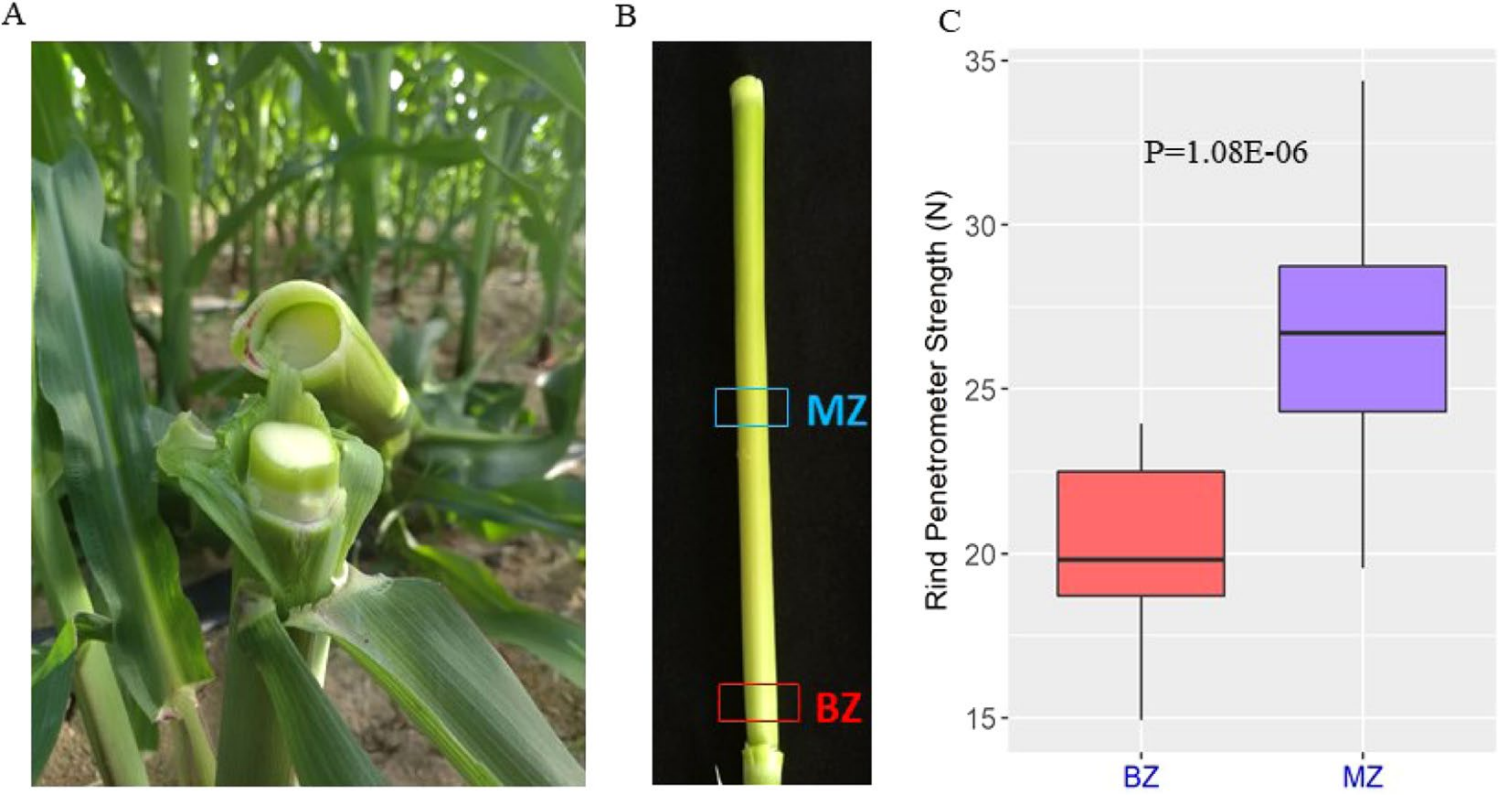
Stalk tissue positions and their mechanical strengths. (**A**) The fracture position of the internode was at the BZ before tasseling. (**B**) The positions of the BZ and MZ. BZ was at 1 cm above the node, with a length of about 1 cm. MZ was at the middle of the internode, also with a length of about 1 cm. (**C**) The rind penetrometer strengths of the BZ and MZ before tasseling. The sample sizes were 20 for the BZ and MZ.

The first internode above the ear of maize inbred line JING724 broke when it was subjected to strong wind stress before tasseling (Fig. 1A), and breaks only occurred at the basal zone (BZ, 1 cm above the node), not the middle zone (MZ), of the internode (Fig. 1B). In addition, the average length of the first internode above the ear of JING724 was 18.66 cm at this stage (Supplementary Fig. S2).

The average rind penetrometer strengths were 19.96 N and 26.47 N for the BZ and MZ, respectively (Fig. 1C), representing a significant difference (p = 1.08E-06) in the mechanical strength of the two positions. However, during the reproductive stage, such as one week after the silking stage, the stalk was stronger and did not break, as the rind penetrometer strength for the BZ and MZ were 34.98N and 35.04N, respectively, with no significant difference (Supplementary Fig. S3). Thus, the subsequent study was only performed for the stage before tasseling.

### Vascular bundle characteristics

**Figure 2:**
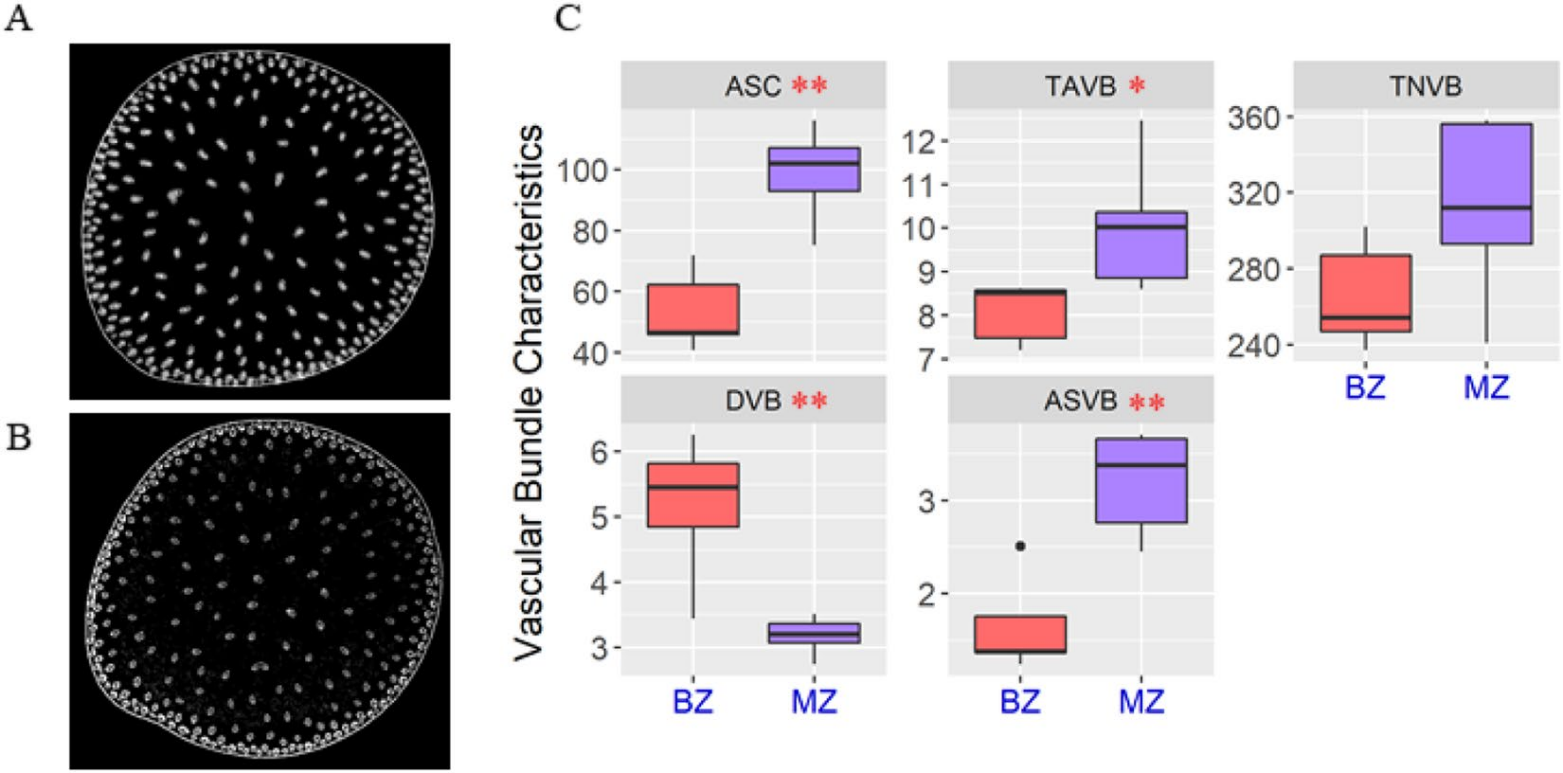
X-ray microcomputed tomography images and vascular bundle traits of the BZ and MZ tissues. (**A**) X-ray microcomputed tomography image for the BZ. (**B**) X-ray microcomputed tomography image for the MZ. (**C**) The distributions and differences of five traits related to vascular bundles for the BZ and MZ, respectively. TNVB: total number of vascular bundles; ASC: area of the stalk cross section; DVB: density of vascular bundles; ASVB: area of a single vascular bundle; TAVB: total area of vascular bundles. Five samples were used for the BZ and MZ, respectively. * and ** indicate the significance at the 0.05 and 0.01 levels, respectively.

Using an X-ray microcomputed tomography scan and a vascular bundle feature extraction process, we measured five vascular bundle characteristics of the two zones (Fig. 2A,B). Except for the total number of vascular bundles, four traits showed significant differences (Fig. 2C). The area of the stalk cross section for the BZ was smaller than that of the MZ, and the total number of vascular bundles in the BZ was slightly less than that in the MZ. However, the density of vascular bundles in the BZ was significantly higher than that in the MZ. By contrast, the area of a single vascular bundle in the BZ was smaller than that in the MZ, and the total area of vascular bundles in the BZ was also less than in the MZ.

Considering the dependence of the total number of vascular bundles and the total area of vascular bundles on the stalk cross section, thus the stalk cross section, the density of vascular bundles and the area of a single vascular bundle were considered as three key features of vascular bundle in BZ and MZ.

### Cell wall component differences between the BZ and MZ

**Figure 3:**
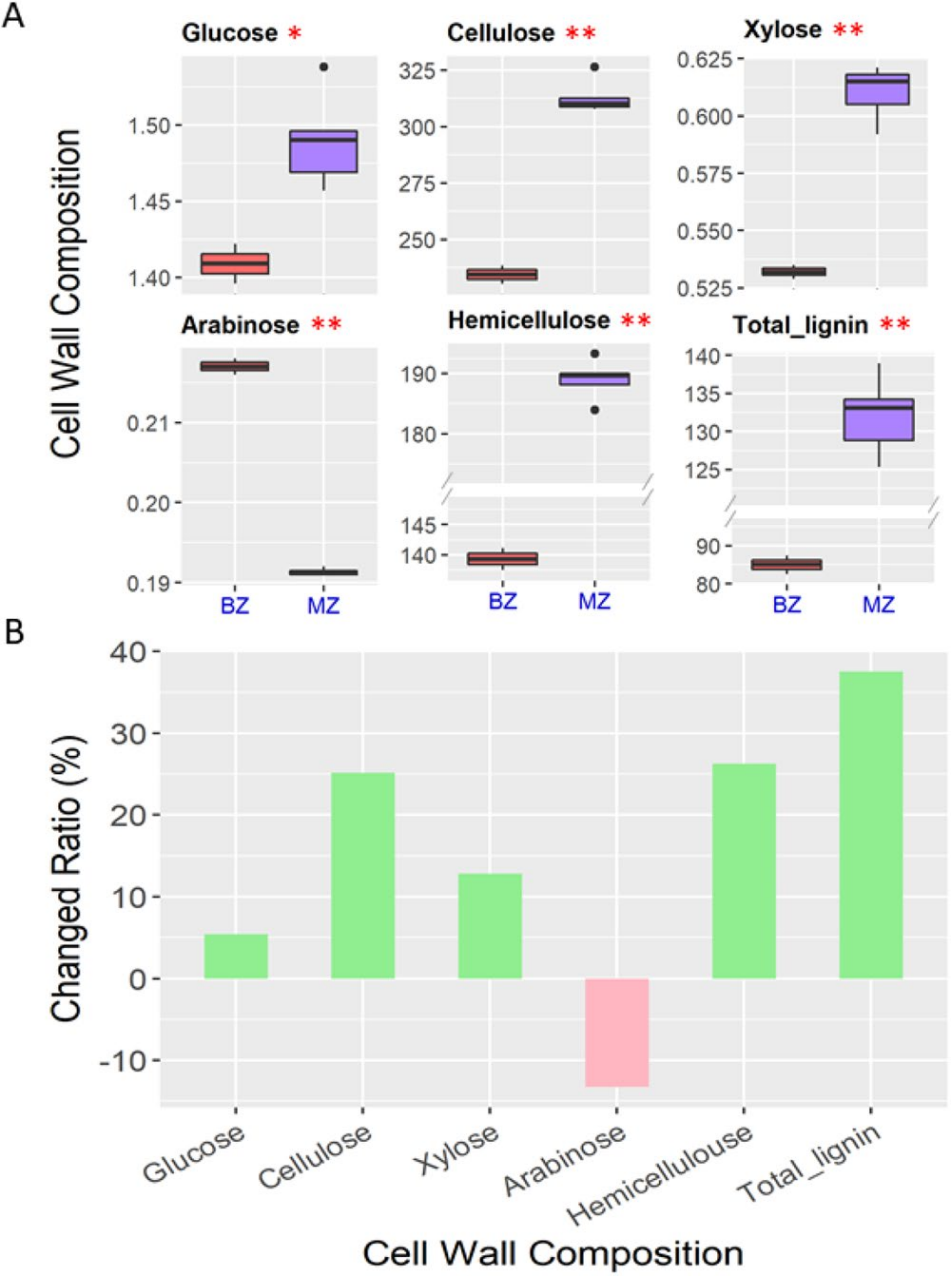
The difference for the six cell wall components for the BZ and MZ. (**A**) Variations and significant differences in six cell wall components. Three biological replicates were performed for each position. * and ** represent the significance at 0.05 and 0.01 level, respectively. (**B**) Increased ratio in the MZ compared to the BZ for the six components. Green bars indicate that the content in the MZ were higher than in the BZ, whereas pink bars indicate the opposite.

Six cell wall components were examined in the BZ and MZ to clarify their distribution gradients. All the components showed significant differences between the two zones. Except for the arabinose, the total amount of the other five component were higher in the MZ than in the BZ (Fig. 3A). Overall, the contents of the main cell wall components in the MZ were greater than those in the BZ. The percentage of the increase in all six cell wall components in the MZ compared to the BZ were calculated to show the rate of changes in the MZ as the development progressesed (Fig. 3B). We can see total lignin was showed 37.52% increase in the MZ compared with that of BZ. Hemicellulose, cellulose, xylose and glucose were also showed increase as 26.30%, 25.12%, 12.82 and 5.43%, respectively (Fig. 3B). In addition, though arabinose showed 13.64% decrease in the MZ compared to BZ, it was accounting for very little portion of the cell wall. Along with the results that rind penetrometer strength of the MZ was higher than that of the BZ, our data indicated that the content of cellulose, hemicellulose, and lignin might be positively correlated to rind penetrometer strength.

### Metabolites related to the BZ and MZ

**Figure 4:**
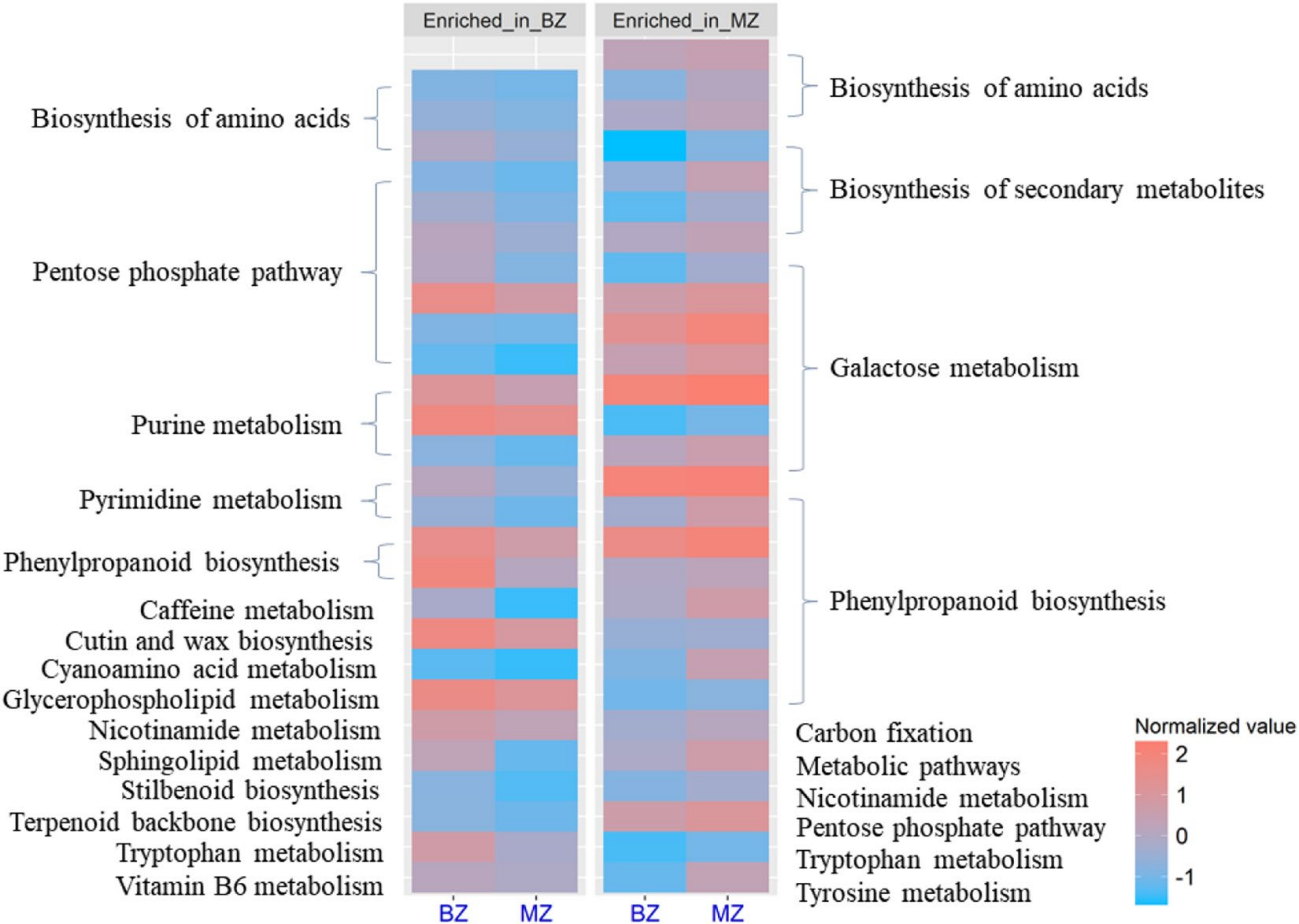
The heatmap and the KEGG annotations for the 27 and 28 metabolites enriched in the BZ and MZ, respectively. Column represents tissues and each row represents the content of the metabolites. The content of metabolites was normalized using the Z-score method on the log10-transformed raw data. Red indicates the metabolite content is high and blue indicates the opposite.

To identify metabolites present during internode development, especially metabolites associated with the internode development gradient, we used liquid chromatography-mass spectrometer to detect metabolites in the BZ and MZ. A total of 2012 metabolites were identified, including 1181 and 831 metabolites under positive and negative ion mode, respectively (Fig. 4; Dataset 1). Among these, 1040 metabolites could be mapped to the primary mass spectrum, 426 metabolites to the secondary mass spectrum, and the remaining 546 metabolites were indistinguishable from the existing mass spectrum.

The orthogonal projections to latent structures-discriminate analysis showed a clear distinction between samples from the BZ and MZ (Supplementary Fig. S4A,B). Based on the criteria of variable importance in the projection >1 and Student’s *t* test P value < 0.05, we identified 399 metabolites with differences in the BZ and MZ, of which 215 were detected under positive ion mode and 184 were detected under negative ion mode. Among them, 174 were enriched in the MZ and 225 were enriched in the BZ (Supplementary Fig. S4C; Dataset 2). In addition, 289 of the total differential metabolites were mapped to the primary or secondary mass spectrum.

We then mapped the 399 differential metabolite between the BZ and MZ to the KEGG database and 55 metabolites were annotated in the KEGG pathways, including 28 and 27 enriched metabolites in the MZ and BZ, respectively. The KEGG pathways contained 28 enriched metabolites in the MZ mainly included secondary metabolite synthesis, such as lignin, phenylpropanoid, and galactose metabolism pathways (Fig. 4). The enriched metabolites in the lignin and galactose pathways were positively related to increases of lignin, cellulose and hemicellulose in the cell wall of the MZ. Twenty-seven enriched metabolites in the BZ were mainly mapped to the pentose phosphate pathway and purine metabolism (Fig. 4).

### Differentially expressed genes in internode development by transcriptomic analysis

To reveal the genes involved in internode development, we performed transcriptome sequencing for the BZ and MZ. High-throughput sequencing generated 21.82–25.69 GB raw reads per sample, 94.4% reached a quality score of Q30, which were mapped to the maize genome B73_V3.25 with a mapping efficiency between 85.34% and 86.91%. A total of 24790 unigenes including 2186 novel genes were generated and annotated to the NR, GO, KOG, and KEGG databases (Dataset 3). PCA results showed that PC1 (explaining 90.6% of the phenotypic variation) well separated the BZ and MZ, indicating that the two sets of samples could be used for transcriptome analysis (Supplementary Fig. S5).

According to the gene expression levels (FPKM values), the reliability of the DEGs (differential expressed genes, abs(log_2_(fold change)) > 1, FDR < 0.01) was analyzed by evaluating the expression differences between the BZ and MZ. In total, 8468 DEGs were identified between the BZ and MZ, among which 4438 and 4030 exhibited higher expression in the MZ and BZ, respectively (Supplementary Fig. S6; Dataset 4).

### Highly expressed DEGs in the MZ

**Figure 5:**
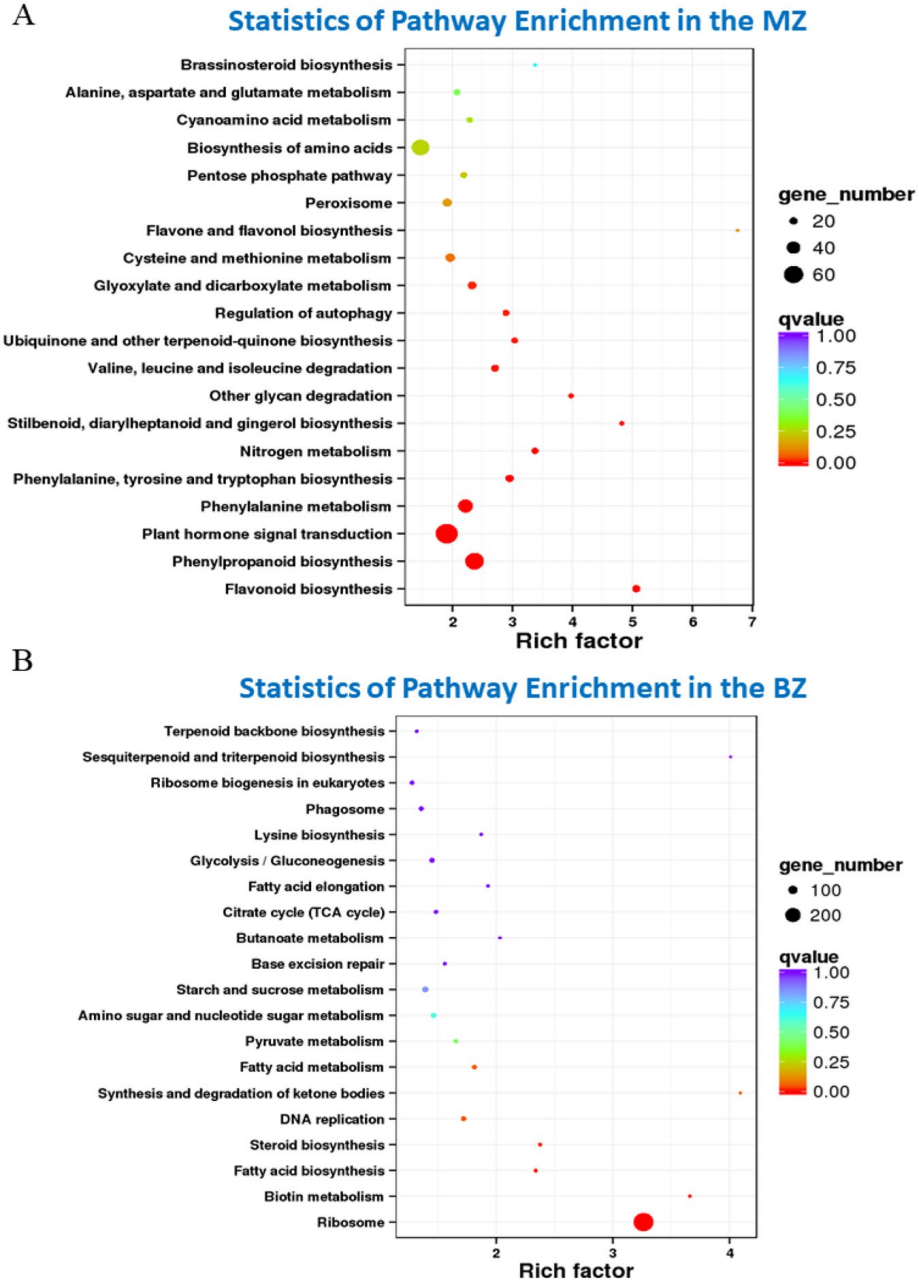
KEGG enrichment of DEGs that were highly expressed in the BZ and MZ. (**A**) KEGG enrichment for the MZ. (**B**) KEGG enrichment for the BZ. The Y-axis represents the pathway name, and the X-axis indicates the rich factor which showes the proportion of DEGs annotated to a pathway compared to the proportion of all the genes annotated to the pathway. The larger the rich factor is, the more significant of enrichment of the DEGs in this pathway is. The color of the circle represents q value which is the P value after correction by multiple hypothesis test. The smaller the q value is, the more reliable the enrichment significance of the DEGs in the pathway is. The size of the circle indicates the number of genes enriched in the pathway, and the color of the dot indicates the significance level.

We annotated the 4438 DEGs highly expressed in the MZ using the KEGG database, and showed that these genes were enriched in hormonal signal transduction pathways and secondary metabolite synthesis, especially phenylpropanoid, phenylalanine, flavonoid, and cellulose-related carbohydrate metabolism pathways (Fig. 5A). The annotation results from the KOG database enrichment analysis were consistent with the KEGG results (Supplementary Fig. S7A).

The DEGs in the hormone transduction system indicated that five kinds of hormones showed greater numbers of highly expressed DEGs in the MZ than those in the BZ, including abscisic acid, ethene, indole acetic acid, zeatin, and salicylic acid (Supplementary Fig. S8).

**Figure 6:**
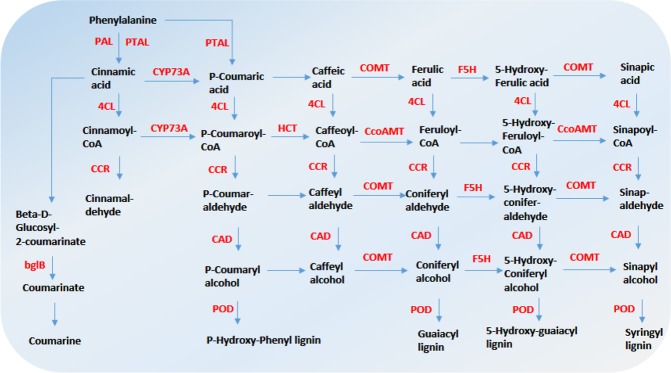
A simplified phenylpropane pathway in the MZ. The phenylpropanoid pathway is the key source for the biosynthesis of lignin. Started from phenylalanine, lignin biosynthesis proceeds via a series of side-chain modifications, ring hydroxylations and O-methylations to produce the lignin monomers. The red font indicates genes that were highly expressed in the MZ. Genes that were not the DEGs or not highly expressed in the MZ in the pathway were not displayed.

Genes related to phenylalanine, phenylpropanoid, and lignin metabolism processes were highly expressed in the MZ, which was consistent with the metabolite and cell wall composition results. In the phenylpropanoid metabolism pathway, the DEGs up-regulated in the MZ invloved in various processes from its initial substrate to final products (Fig. 6), including key enzymes in the process of lignin synthesis: phenylalanine ammonia-lyase (PAL), 5-hydroxylase (F5H), coenzyme A ligase (4CL), hydroxycinnamoyl-CoA reductase (CCR), hydroxycinnamic acid dehydrogenase (CAD) and peroxidase (POD).

To validate the expression profiles of the DEGs, six DEGs in the phenylpropanoid pathway were selected for qRT-PCR, including *pal2* (PAL), *Zm4CL* (4CL), *ZmCAD* (CAD), *cncr2* (CCR), *ZmCOM* (COMT), and *pox3* (POD) (Supplementary Table S1). The qRT-PCR results for the six DEGs were similar to those obtained by transcriptomic analysis (Supplementary Fig. S9), implying that the DEGs identified by RNA-seq were reliable.

### Highly expressed DEGs in the BZ

The DEGs upregulated in the BZ mainly enriched in ribosome, fatty acid, and sterol synthesis (Fig. 5B; Supplementary Fig. S7B). There were 587 genes involved in the composition of the ribosome, among which 273 DEGs were highly expressed in the BZ, suggesting the vigorous protein synthesis in the BZ as ribosomes are mainly responsible for the synthesis of intracellular proteins. Consistent with the increase in ribosome pathway genes, DEGs involved in the DNA replication pathway were also highly expressed in the BZ, in which components of the DNA polymerase alpha-primase complex, the delta primer complex and TCT were significantly elevated, indicating that the process of DNA replication is highly active (Fig. 5B). In addition, 21 and 22 highly expressed DEGs were identified in sterol and fatty acid pathways in the BZ, respectively (Fig. 5B; Supplementary Fig. S10).

## Discussion

The development of the plant stalk is spatially and temporally specific, and significant differences are observed among different internodes of the same individual and even among different parts of the same internode. Field observation has showed that stalk breakage often occurs at the BZ when subjected to strong wind before tasseling. The average length of the first internode above the ear of JING724 was 18.66 cm (Supplementary Fig. S2). Therefore, according to Scobbie *et al*. (1993) and Zhang *et al*. (2014), the BZ (fracture position) is at the first section, and the MZ position is at the sixth section, which belong to the meristem zone and mature zone, respectively^16,19^.

We systematically compared the phenotypes, from the macroscopic to the microscopic scale, of the BZ and MZ, and explored the types of genes that affect these phenotypes (Supplementary Fig. S11).

For the MZ, the rind penetrometer strength was significantly higher than that of the BZ (Fig. 1), which explained its stronger than BZ. In order to dissect the mechanisms underlying the difference in mechanical strength, we measured the stalk cell wall components, which indicated that the level of cellulose, hemicellulose and lignin contents of the MZ were significantly higher than that of the BZ (Fig. 3). This is consistent with the previous studies in which the stalk strength was proportional to the cellulose and lignin contents of the cell wall^20,21^.

The recently developed metabolomic methods can detect thousands of metabolites in cells and have many applications in crop yield analysis^22^. However, such methods have not yet been applied to the study on stalk development. It was demonstrated that 28 known metabolites enriched in the MZ can be mapped to the lignin, phenylpropanoid and galactose metabolism (Fig. 4). Phenylpropanoid pathway is the main pathway for lignin synthesis^10^, and galactose metabolism can produce glucose, xylose and mannose, which are the main components of cellulose and hemicellulose^4,7,8^, so such results were consistent with the higher contents of cellulose, hemicellulose, and lignin in the MZ. The 4438 up-regulated DEGs in the MZ were mainly involved in secondary metabolites sysnthesis, such as phenylpropanoid, lignin and hormone metabolism pathways (Fig. 5A). Previous studies have showed that mutations in phenylpropanoid pathway, such as *bm1*, *bm2*, *bm3* and *bm4*, resulted in the variation in the lignin and stalk mechanical strength^23–26^. Overall, all data of the transcriptomic, metabolomic, and phenotypic profiling suggested that secondary metabolite synthesis was very active in the MZ, so the deposition of secondary cell wall compenents and thus, the lignin level in MZ was greater than that of the BZ.

The rind penetrometer strength of BZ, the stalk break spot, was weaker than that of the MZ. The cellulose, hemicellulose and lignin contents of the BZ were significantly less than that of the MZ. In addition, with the simple and fast X-ray microcomputed tomography techniques through scanning the entire stalk cross section area, we observed that the density of vascular bundles was higher but their sizes were smaller in the BZ compared to the MZ, suggesting that the BZ may be responsible for early differentiation of vascular bundles (Fig. 2). The 27 known metabolites were mapped to pentose phosphate pathway and purine metabolism whose main physiological function were to produce energy (Fig. 4)^27^. We speculate that cells in the BZ requires a large amount of energy to synthesize more substances. The 4030 highly expressed DEGs in the BZ were mainly involved in ribosome synthesis, suggesting that many proteins are being synthesized (Fig. 5B). In addition, the genes in sterol pathway which contributes to the synthesis of brassinosteroid were also highly expressed^28^. Ibañes *et al*. (2009) have shown that brassinosteroid can regulate the number of vascular bundles in the shoot by promoting the division of cells in the original layer^29^. Therefore, the large amount brassinosteroid synthesis in the BZ tissue may be related to the differentiation of vascular bundles, and may also explain the higher density of vascular bundles in the BZ. To summarize, extensive protein synthesis and vascular bundle synthesis were highly active in the BZ, whereas the MZ, on the contrary, was a relatively well developed region on the stalk with substantial secondary cell wall deposition.

**Figure 7:**
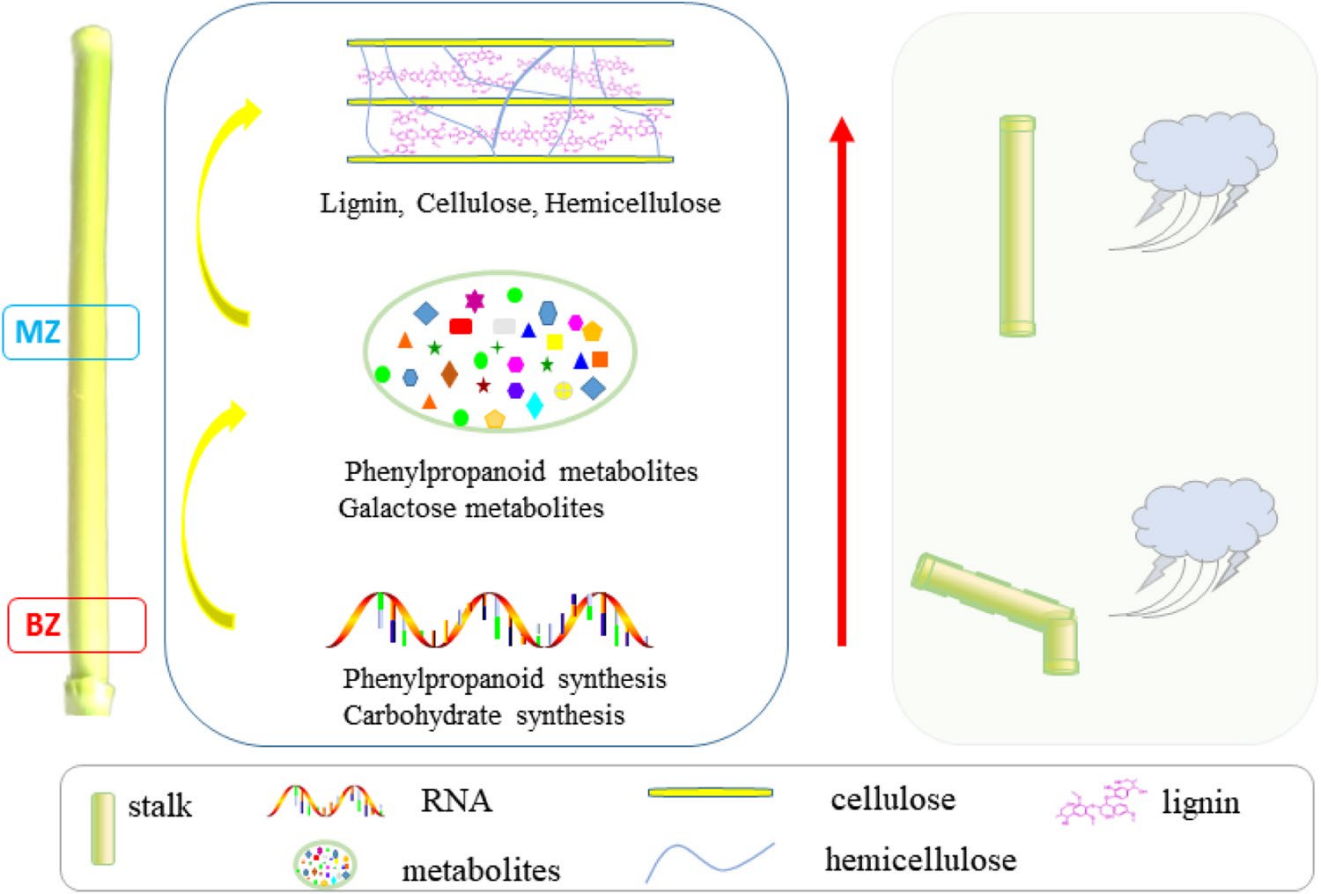
An integrated model that depicts the relationship between internode development and stalk fracture resistance. From the BZ to MZ, the synthesis of phenylpropanoid and carbohydrates were significantly increased, the corresponding metabolites such as phenylpropanoid and galactose were increased, and lignin, cellulose and hemicellulose in the cell wall were also increased. A proposed solution to solve the BZ fracture was to increase the synthesis of phenylpropane and carbohydrate in the BZ region, and promote the deposition of the secondary cell wall.

Before tasseling, the stalk was easily broken at the BZ, which is mainly due to its rapid vegetative growth and the less secondary cell wall synthesis in this stalk section, resulting in a weak stalk. After entering the reproductive growth stage, the stalk was stronger and not easy to break (Supplementary Fig. S3). Based on the results of multi-omics experiments, we have depicted a model to explain the relationship between stalk fracture resistance and internode development and proposed potential solutions to improve the stalk mechanical strength before tasseling (Fig. 7). We found that from the BZ to MZ, the synthesis of phenylpropanoid and carbohydrates was significantly increased at the transcriptional level. The corresponding metabolites such as phenylpropanoid and galactose metabolism, as well as the cell wall component, such as lignin, cellulose and hemicellulose, were also increased from the BZ to MZ. The systematical comparison indicated that the main reason of stalk fragile in BZ was less accumulation of the secondary metabolites related to secondary cell wall compared to the MZ. Therefore, slowing down the longitudinal elongation of the internode at this stage to increase the deposition of its secondary cell wall could be an effective approach to improve BZ fracture resistance. For example, in the agronomic practice of maize, applying plant growth regulators during jointing stage can shorten the internode length but increase the internode diameter and the thickness of the cell wall, especially the development of the secondary cell, thereby reducing the risk of stalk lodging^30^. In maize agronomic practice, spraying plant growth regulators on the hybrid of this material (Jingke968) during jointing stage can significantly increase the diameter of the internodes and reduce the rate of stalk fracture (data unpublish). Thus, our results may guide the field management practice to reduce lodging in the field, and more importantly, provide the theoretical basis for the breeding of maize varieties with more secondary cell wall deposition in the stalk to avoid lodging.

## Materials and Methods

### Plant materials

The commercial inbred line JING724 was obtained from the Maize Research Center, Beijing Academy of Agriculture and Forestry Sciences, China. Plants were grown at the Hainan field station in the winter of 2017. Before tasseling, the first internode above the ear was collected, and the BZ (1 cm above the node, with a length of about 1 cm) and MZ (the middle of the internode, with a length of about 1 cm) parts were sampled for subsequent investigation (Fig. 1B).

### Internode length and mechanical strength measurement

Before tasseling, 17 uniform plants were selected to measure the length of the internode by ruler, and 20 samples of the BZ and MZ parts, respectively, were used for the mechanical strength measurement (Supplementary Table S2). In addition, one week after silking (about two weeks from the first measurement), we also measured the mechanical strength for 16 samples of the BZ and MZ parts, respectively (Supplementary Table S2). The rind penetrometer strength for the BZ and MZ was measured with an electronic penetrometer (YYD-1, Zhejiang Top Cloud-Agri Technology Co., Ltd., Zhejiang, China). It mainly uses a pressure sensor to detect the puncture strength of the rind, and the probe is a pointed type with a diameter of 1 mm. The maximum instantaneous force that penetrated the rind was recorded as the rind penetrometer strength of the internode. Student’s *t*-test was used to identify significant differences.

### Identification of vascular bundles based on X-ray microcomputed tomography

Stalk samples from the BZ and MZ were collected, each with 5 replicates (Supplementary Table S2). Then, the vascular bundle characteristics were determined according to Du *et al*. (2016) and Zhang *et al*. (2018) through the following steps^31,32^. (1) The sample was soaked in FAA solution (90:5:5 v/v/v, 70% ethanol: 100% formaldehyde: 100% acetic acid) for at least 3 days until all the tissues were infiltrated. (2) The samples were dehydrated with a series of concentrations of ethanol at 30, 50, 70, 85, 95 and 100%, 30 min for each step. (3) The samples were dried using a CO_2_ critical-point drying system. (4) The samples were dyed with volatile iodine vapor. (5) Micro-CT scanning was performed using the Skyscan 1172 X-ray microcomputed tomography system (Bruker Corporation). (6) Images were acquired and three-dimensional reconstruction of vascular bundles was conducted. The automatic imaging software CTAn and CTVol (Bruker Corporation) was used to reconstruct three-dimensional surface models of the vascular bundles. Five vascular bundle traits were extracted, including the area of the stalk cross section (units of mm2), total area of vascular bundles (units of mm2), total number of vascular bundles, density of vascular bundles, and area of a single vascular bundle (units of mm2; with total area of vascular bundles divided by total number of vascular bundles). Statistical significance were determined using the Student’s *t*-test.

### Detection of stalk cell wall components

A total of 30 samples were collected from both the BZ and MZ. The samples were inactivated at 105 °C for half an hour and then transferred to an 80 °C oven for drying.

Ten dried samples of the same tissue were pooled, grounded and screened through a combined 40–80 mesh sieve. The structural carbohydrates (i.e., glucose, xylose, and arabinose) and lignin (acid-soluble lignin and acid-insoluble lignin) were extracted using a two-step sulfuric acid hydrolysis process. Sample quantity was measured using a high performance liquid chromatography system (1260 series, Agilent Technologies, Santa Clara, CA, USA)^33^. The cellulose content was mainly calculated from the glucose content. Hemicellulose was determined by xylose and arabinose contents. Lignin was calculated as the sum of the acid-soluble and acid-insoluble lignin contents. The unit of glucose, xylose and arabinose were mg/ml; and the unit of cellulose, hemicellulose and lignin were g/kg. The increased ratios of these components in the MZ compared with the BZ were calculated using the increment in the MZ divided by the amount of the BZ. Student’s *t*-test was performed for the statistical significance.

### Metabolite detection and analysis

Nine BZ and six MZ samples were collected and frozen using liquid nitrogen. Three samples from the same tissue were combined as a mixed sample. Thus, there were three and two mixed samples for the BZ and MZ, respectively. Metabolite extraction and analysis were performed using a liquid chromatography-mass spectrometer^34^. The metabolite detection process was as follows: An Agilent 1290 ultra high performance liquid phase was used to control the mobile phase parameters. The AB 5600 Triple TOF mass spectrometer was capable of performing primary and secondary mass spectrometry data acquisition based on the IDA function under the control of the control software (Analyst TF 1.7, AB Sciex). In each data acquisition cycle, the molecular ion with the strongest intensity greater than 100 was selected to collect the corresponding secondary mass spectrometry data. Bombardment energy: 30 eV, 15 secondary spectra every 50 ms. The ESI ion source parameters were set as follows: atomization pressure (GS1): 60 Psi, auxiliary pressure: 60 Psi, air curtain pressure: 35 Psi, temperature: 650 °C, spray voltage: 5000 V (positive ion mode) or −4000 V (negative ion mode)^35^.

Raw MS data were converted to the mzXML format using ProteoWizard, and processed with the R package XCMS (version 3.2)^36^. Then, the R package CAMERA was used for peak annotation. An in-house MS2 database was used for metabolite identification. We calculated the fold change of metabolites between BZ and MZ. For the identification of differential metabolites, we used latent structures-discriminate analysis (OPLS-DA) together with Student’s *t* test^37,38^. OPLS-DA has the advantage of filtering out the orthogonal variables in the metabolite that was not related to the categorical variables then obtaining more reliable metabolite information on the differences between groups^39^. OPLS-DA was performed using ropls package in R 3.3.2. The effectiveness of the model can be reflected from R^2^X, R^2^Y and Q^2^, where R^2^X and R^2^Y represent the explanation rate of the model for X and Y matrix, and Q^2^ represents the prediction ability of the model. The closer of the three parameters are close to 1, the more stable and reliable the model. To validate the reliability of the OPLS-DA model, we also performed permutation tests. For the effective OPLS-DA model, differential metabolites were preliminary screened according to variable importance in the projection (VIP) analysis. Finally, the differential metabolites were selected by combining the P value of the Student’s *t* test and the VIP value of the OPLS-DA model. Only the metabolites whose P value < 0.05 and VIP > 1 can be refered as differential metabolites^37,38^.

To analyze the function of the differential metabolites, we mapped them to the KEGG database (http://www.kegg.jp/kegg/pathway.html) to screen the significant enriched pathways of the differential metabolites based on enrichment analysis and hierarchical clustering analysis using KOBAS2.0 software^40–43^.

### Transcriptome sequencing and data analysis

The samples used for RNA sequencing and analysis were the same as those used for metabolite detection. RNA was extracted following the standard protocol of the Quick RNA isolation Kit (Huayueyang Biotechnology Co., Ltd. Beijing, China). The RNA concentration was measured using a NanoDrop 2000 (Thermo). RNA integrity was assessed using the RNA Nano 6000 Assay Kit of the Agilent Bioanalyzer 2100 system (Agilent Technologies). Sequencing libraries were generated using NEBNext Ultra™ RNA Library Prep Kit for Illumina (NEB, USA) following the manufacturer’s recommendations, and index codes were added to attribute sequences to each sample. The libraries were sequenced using an Illumina HiSeq™ 2000 at the Beijing Genomics Institute (BGI). Low-quality sequence reads were removed from the data sets. The clean reads were then mapped to the reference genome (B73 AGPv3.25 reference) using the Hisat2 tools software.

Unigene annotation was performed using the BLAST software^44^. Gene functions were annotated based on the following databases: COG (Clusters of Orthologous Groups of proteins for Prokaryotes, http://www.ncbi.nlm.nih.gov/COG/), GO (Gene Ontology, http://www.geneontology.org/), KEGG (Kyoto encyclopedia of Genes and Genomes, http://www.genome.jp/kegg/)^41–43^, KOG (Clusters of Orthologous Groups of proteins for Eukaryotes, http://www.ncbi.nlm.nih.gov/KOG/), NR (NCBI non-redundant protein sequences, http://ftp.ncbi.nih.gov/blast/db/), Pfam (Protein family, http://pfam.xfam.org/), and Swiss-Prot (a manually annotated and reviewed protein sequence database, http://www.uniprot.org/). KOBAS2.0 was used to test the statistical enrichment of differential expression genes in KEGG pathways^40^. Gene expression levels were estimated by fragments per kilobase of transcript per million fragments mapped (FPKM) values^45^. Differential expressed genes (DEGs) between two tissues were obtained using the DESeq R package (1.10.1)^46^. The resulting P-values were adjusted using Benjamini and Hochberg’s approach for controlling the false discovery rate (FDR)^47^. Genes with an abs(log_2_(fold change)) > 1 and FDR < 0.01 were considered as the DEGs.

### Real-Time quantitative qRT-PCR

The RNA samples used for qRT-PCR were the same as those used for transcriptome sequencing. The cDNA was synthesized using a PrimeScript™ RT reagent kit with gDNA Eraser (TaKaRa, RR047A). The primers used for qRT-PCR were synthesized at the Beijing Invitrogen Company (Supplementary Table S1). The PCR mixture was 2 × Master Mix (10 µl), 10 μM F and R primers (0.5 µl each), and water to make the total volume of 20 µl. The PCR program was set as 95 °C, 30 sec; 40 cycles (95 °C, 5 sec; 60 °C, 40 sec (fluorescence collected)). Melting curves were established for the PCR products at the end of the amplification reaction (95 °C, 10 sec; 60 °C, 60 sec; 95 °C, 15 sec) with slow heating from 60 °C to 99 °C (the instrument automatically ramped the temperature at a rate of 0.05 °C/sec). The target gene and the actin gene of each sample were subjected to the real-time PCR reaction, and each sample was tested in three replicate wells. The level of target genes was calculated using the relative ΔΔCt method^48^.

## Supplementary information

Multi-omics analysis of the development and fracture resistance for maize internode

Dataset 1

Dataset 2

Dataset 3

Dataset 4
