# Supplementary material for: Multi-omics analysis of the development and fracture resistance for maize internode
Source: Sci Rep. 2019 Jun 3;9:8183. doi: 10.1038/s41598-019-44690-6 (PMC6547879; doi:10.1038/s41598-019-44690-6)
Supplement: Supplementary file 1 — Multi-omics analysis of the development and fracture resistance for maize internode [file 41598_2019_44690_MOESM1_ESM.docx]

**SUPPORTING INFORMATION**

**Multi-omics analysis of the development and fracture resistance for maize internode**

Xiaqing Wang^1,3^, Ruyang Zhang^1,3^, Zi Shi^1^, Ying Zhang^2^, Xuan Sun^1^, Yulong Ji^1^, Yanxin Zhao^1^, Jidong Wang^1^, Yunxia Zhang^1^, Jinfeng Xing^1^, Yuandong Wang^1^, Ronghuan Wang^1^, Wei Song^1,*^, Jiuran Zhao^1,*^

^1^Beijing Key Laboratory of Maize DNA Fingerprinting and Molecular Breeding, Maize Research Center, Beijing Academy of Agriculture and Forestry Sciences, Shuguang Huayuan Middle Road, Haidian District, No. 11, Beijing 100097, China.

^2^Beijing Key Lab of Digital Plant, Beijing Research Center for Information Technology in Agriculture, Beijing Academy of Agriculture and Forestry Sciences, Shuguang Huayuan Middle Road, Haidian District, No. 11, Beijing 100097, China.

^3^These authors contributed equally to this work.

^*^Correspondence should be addressed to

Wei Song, [songwei1007@126.com](mailto:songwei1007@126.com), Tel: (+86) 01051503983

Jiuran Zhao, [maizezhao@126.com](mailto:maizezhao@126.com), Tel: (+86) 01051503936


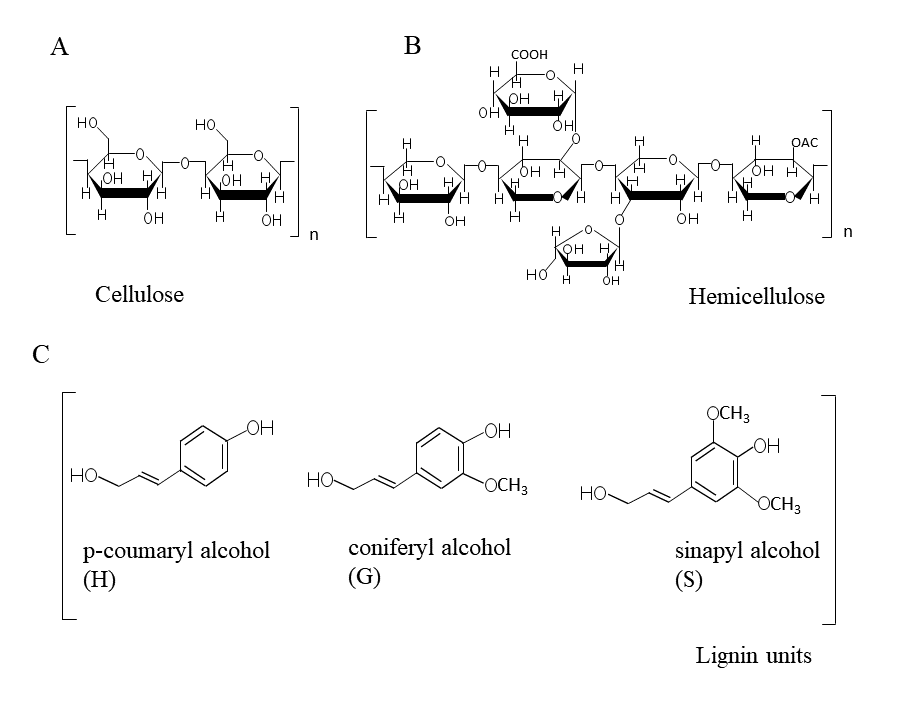


**Supplementary Figure S1.** **The structure of cellulose, hemicellulose and lignin.**

(**A**) The structure of cellulose. The picture was modified from Qiu & Hu. (2013)^4^. (**B**) The structure of hemicellulose. The picture was modified from Deshavath (2017)^7^. (**C**) Three lignin monomers. The picture was modified from Picart (2015)^11^.


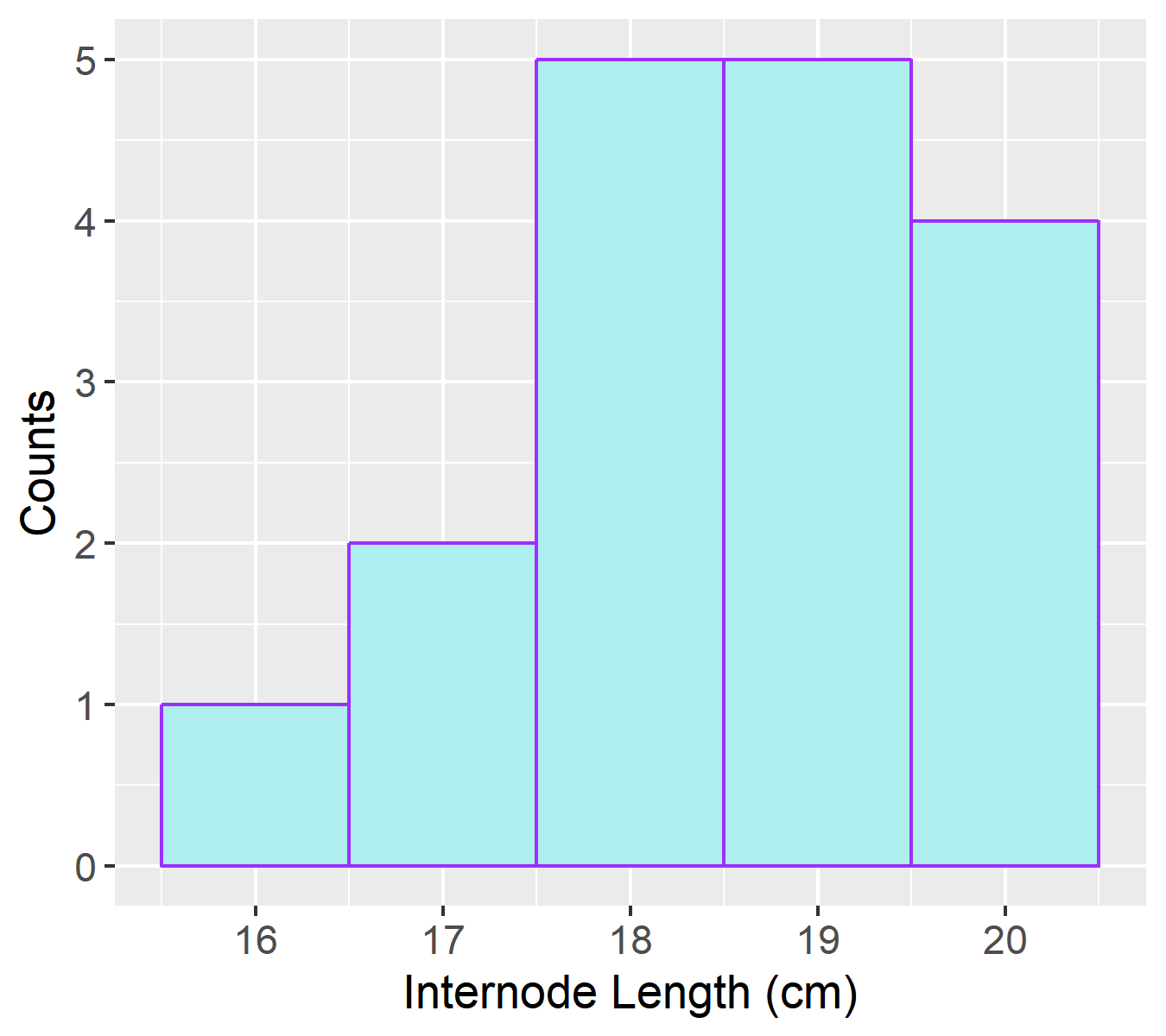


**Supplementary Figure S2.** **The average length of the first internode above the ear of JING724.**

The X-axis represents the length of internode and the Y-axis represents the number of individuals.


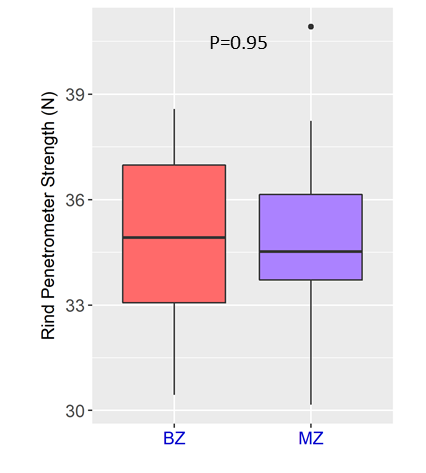


**Supplementary Figure S3. The rind penetrometer strength of the BZ and MZ at one week after silking stage.**

The rind penetrometer strength of the BZ and MZ were 34.98 N and 35.03 N, respectively, with sample size were 16 for each. There and no significant difference for the rind penetrometer strength.


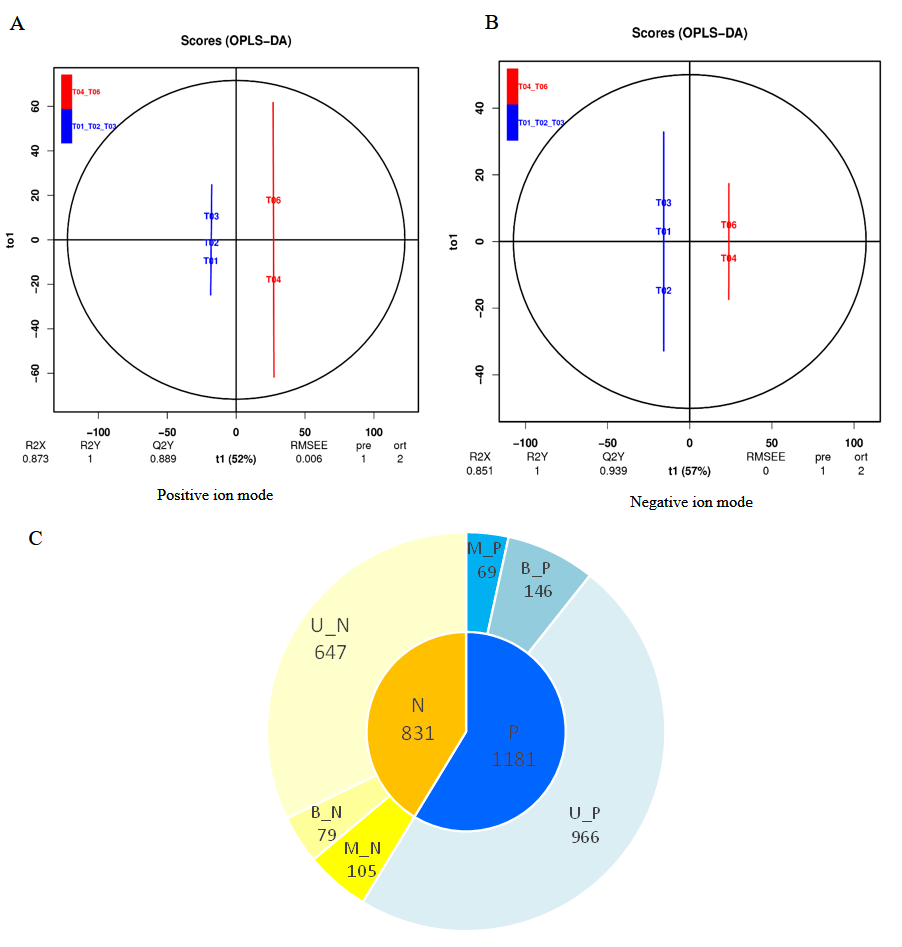


**Supplementary Figure S4.** **OPLS-DA plot and the number of metabolites detected in positive and negative ion, respectively.**

(**A**) The OPLS-DA plot based on positive ion mode. The R^2^X, R^2^Y and Q^2^Y was 0.873, 1 and 0.889, respectively. (**B**) The OPLS-DA plot based on negative ion mode. The R^2^X, R^2^Y and Q^2^Y was 0.873, 1 and 0.939, respectively. Samples of the BZ and MZ were labeled with blue and red font, respectively. (**C**) Metabolites numbers. The inner circle indicates the total metabolites. The outer circle classifies the metabolites into 399 changed metabolites and 1613 unchanged metabolites. N and P represent metabolites detected in negative and positive ion mode, respectively. B, M are the metabolites enriched in the BZ and MZ, respectively. U means unchanged metabolites.


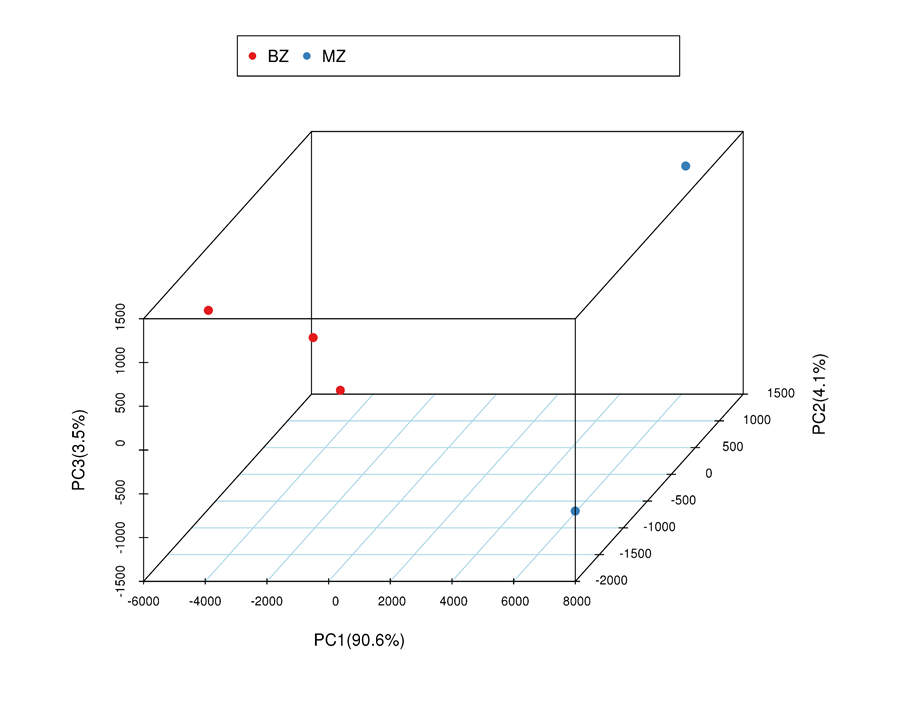


**Supplementary Figure S5.** **PCA plot for RNA-Seq data.**

Samples of BZ and MZ were labeled with blue and red font, respectively.


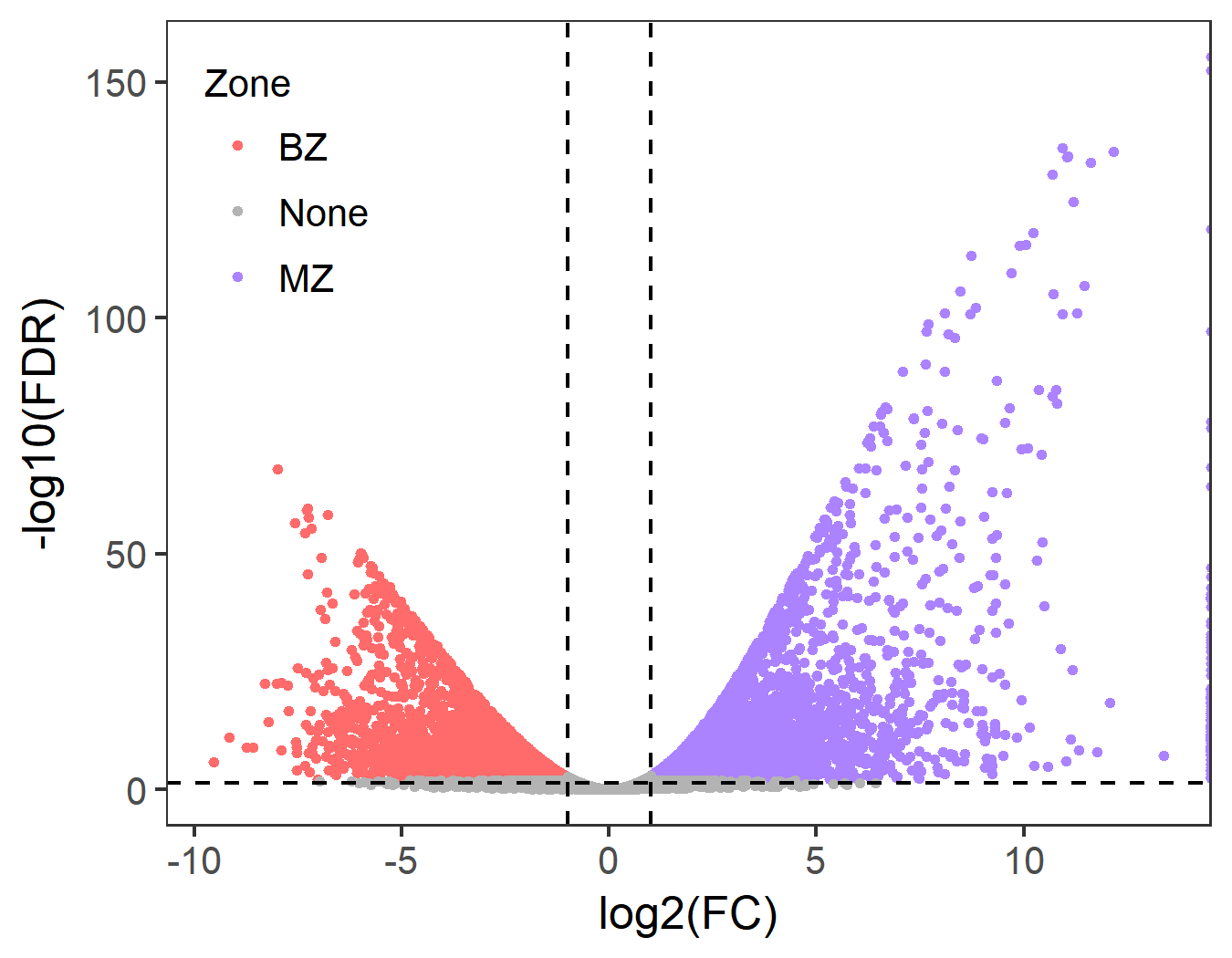


**Supplementary Figure S6**. **DEGs for the BZ and MZ, respectively.**

Red and blue dot represent the genes up regulated in the BZ and MZ, respectively. Grey dots represent the genes with expression unchanged significantly.


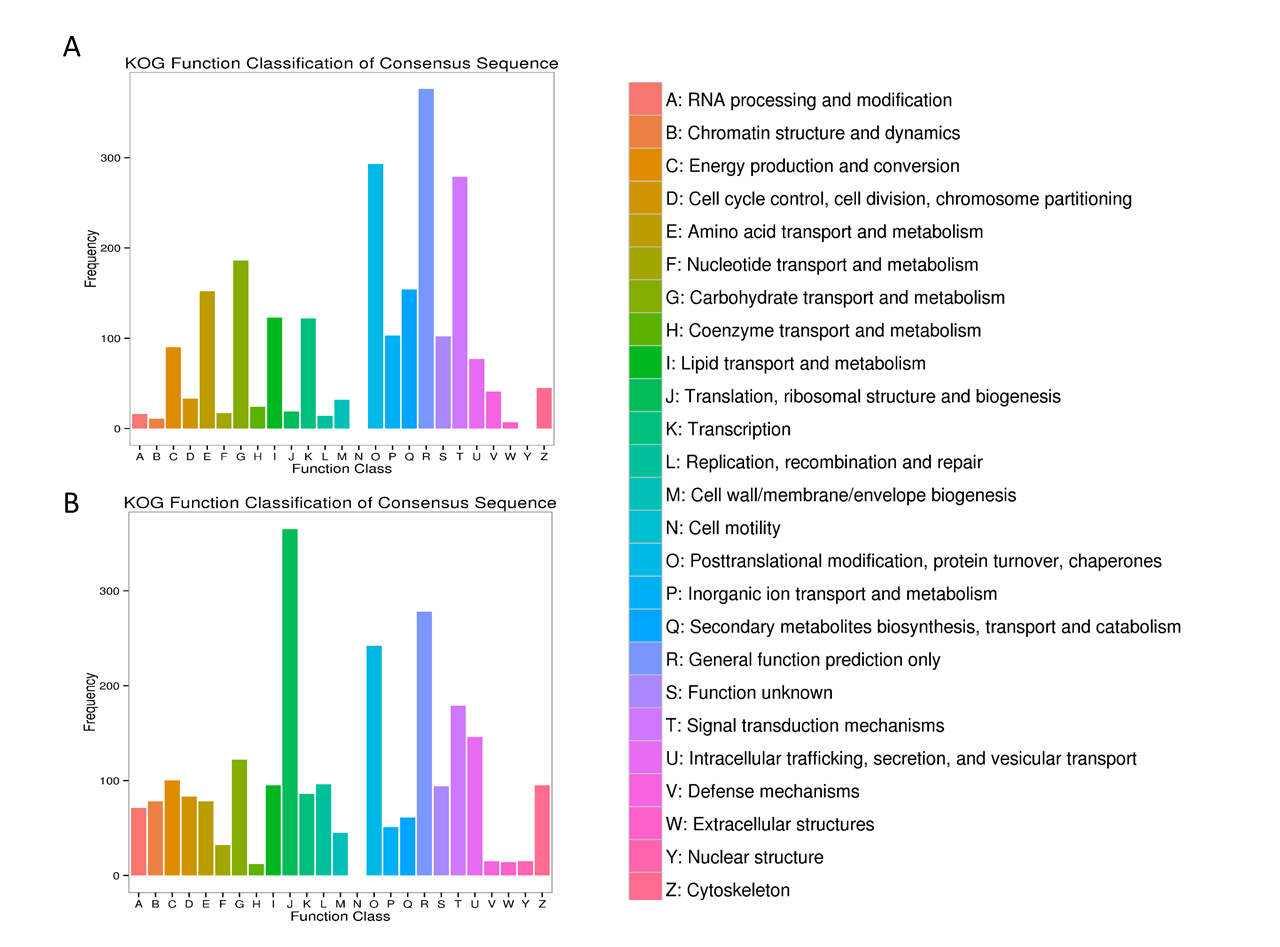


**Supplementary Figure S7.** **KOG analysis for both the BZ and MZ, respectively.**

(**A**) KOG result of the MZ. (**B**) KOG result of the BZ.


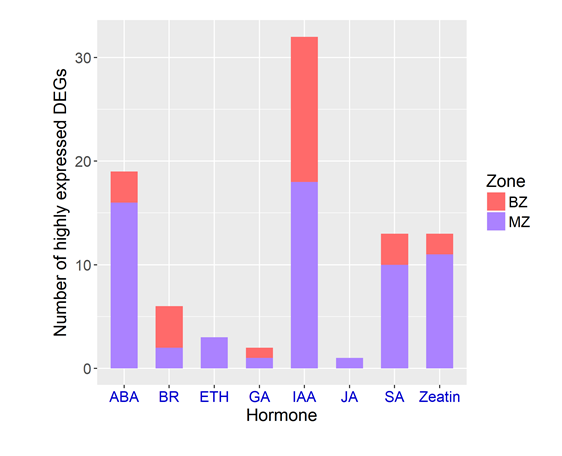


**Supplementary Figure S8.** **Hormone related DEGs in the BZ and MZ, respectively.**

Red and blue indicate the highly expressed genes in the BZ and MZ, respectively. ABA: abscisic acid; BR: brassinosteroid; ETH: ethene; GA gibberellin; IAA: indole acetic acid; JA: jasmonic acid; SA: salicylic acid.


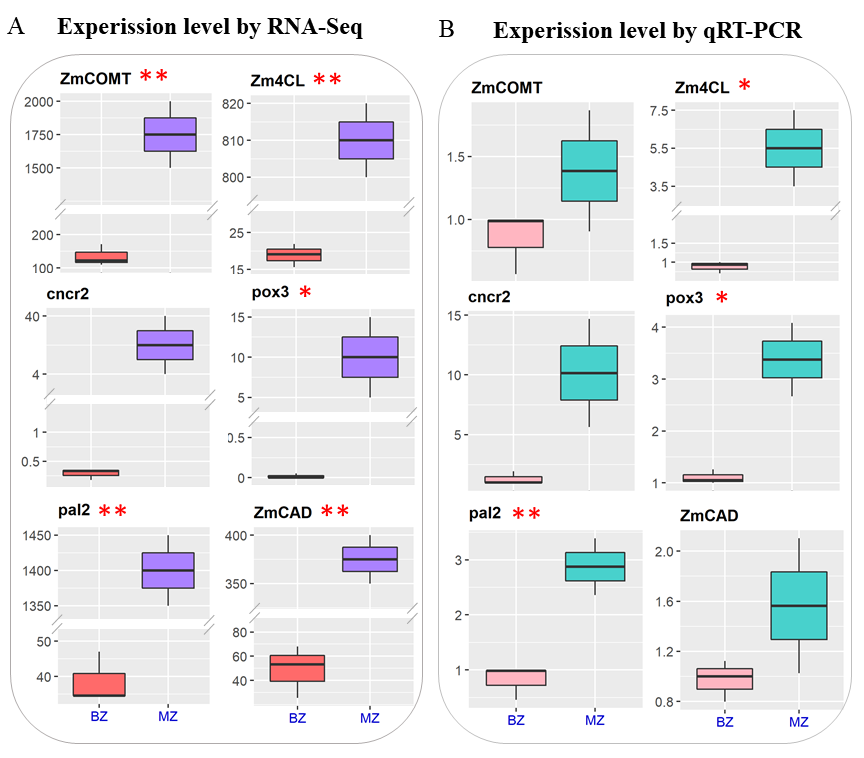


**Supplementary Figure S9.** **Compative expression level by RNA_Seq and qRT-PCR of six genes**.

(**A**) Expression by RNA_seq in BZ and MZ. (**B**) qRT-PCR results of gene expression in B and TZ. Actin was the reference gene. qRT-PCR data calculated with the 2-△△ct method. * and ** indicate at 0.05 and 0.01 significant level, respectively.


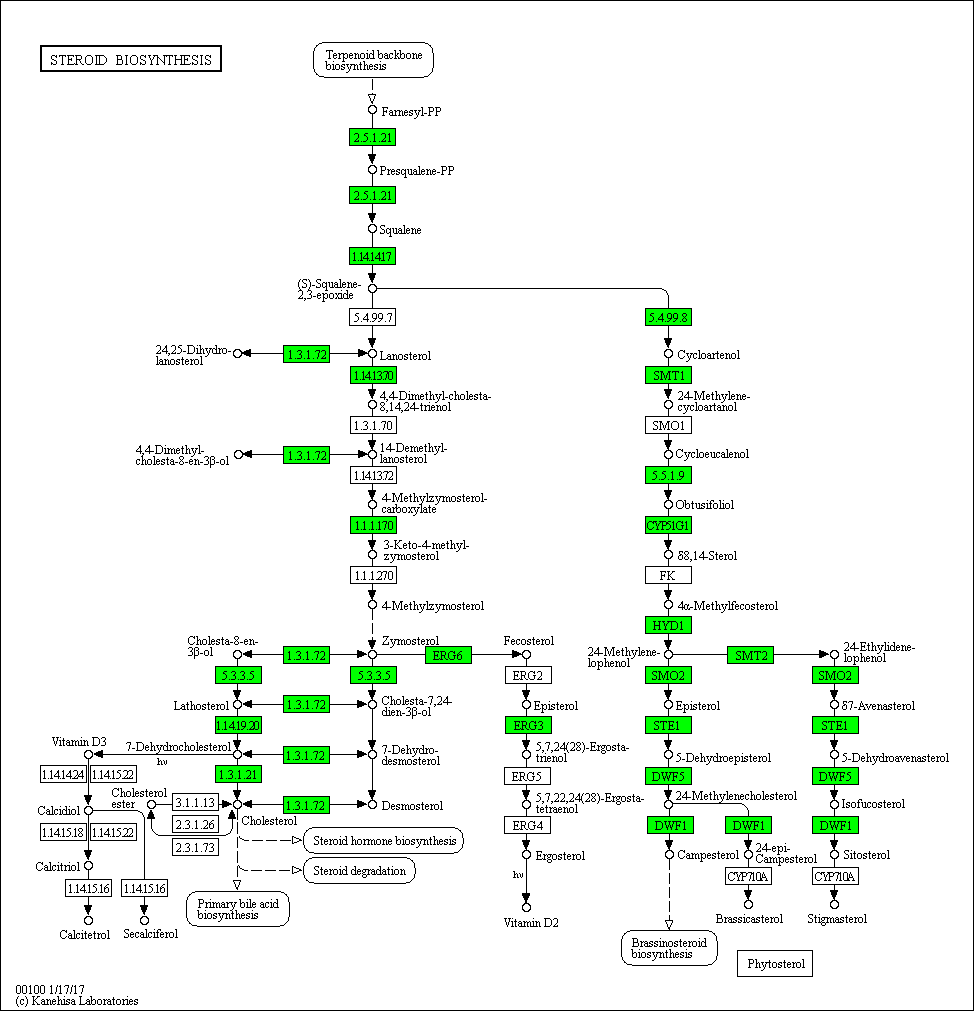


**Supplementary Figure S10.** **KEGG pathway for steroid biosynthesis in the BZ.**

The steroid biosynthesis pathway was cited from map00100 of KEGG database with permission^41-43^. The green marked boxes indicated the genes highly expressed in the BZ.


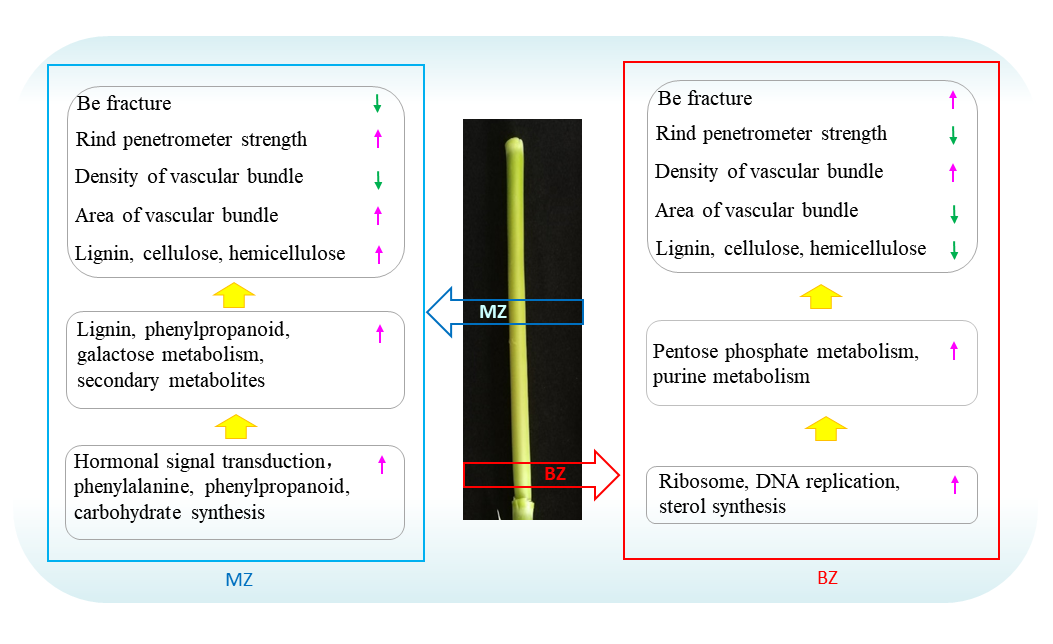


**Supplementary Figure S11.** **A comparison of the main differences between the BZ and MZ of the internode.**

The blue box shows the characteristics of the MZ. The red box shows the characteristics of the BZ. Pink up arrows indicate a larger value or enrichment; green down arrows indicate small values. Larger yellow arrows represent regulation from the transcriptome to the metabolome and the macroscopic phenotype.

**Supplementary Table S1. Primers for qRT-PCR.**

| **Gene ID** | **Gene name** | **F/R** | **5'-3'Primers** |
| --- | --- | --- | --- |
| ACTIN |  | F | GCTATCCAGGCTGTTCTTTCG |
| ACTIN |  | R | CATTAGGTGGTCGGTGAGGT |
| Zm00001d003016 | *pal2* | F | GCCCATCTGCTGAGGAGTGAG |
| Zm00001d003016 | *pal2* | R | GCCTGCTTGAGAATACCGAAATAC |
| Zm00001d015459 | *Zm4CL* | F | AATGAACGATGACCTTGCTGG |
| Zm00001d015459 | *Zm4CL* | R | TCGGTGAAGAAGACCTTGTGG |
| Zm00001d015618 | *ZmCAD(bm1)* | F | CAAGATGGGGTACGTGAACGAG |
| Zm00001d015618 | *ZmCAD(bm1)* | R | AGAAGGCGGAAAGACAACAACT |
| Zm00001d019669 | *cncr2* | F | AGTACCCCGTACCCACCAAG |
| Zm00001d019669 | *cncr2* | R | CGTCTCGTAGAGGCACTGGA |
| Zm00001d037547 | *pox3* | F | CAGACAGGTTCGCCTCCAAG |
| Zm00001d037547 | *pox3* | R | AAGAAATGCGTAGGCTTTGCTC |
| Zm00001d049541 | *ZmCOMT(bm3)* | F | CACCTACATCTACGCCAACGC |
| Zm00001d049541 | *ZmCOMT(bm3)* | R | GAGGACCAAGCAAGCAAAGC |

**Supplementary Table S2. Samples used for each experiments.**

| **Experiments** | **Number of samples for the BZ** | **Number of samples for the MZ** | **Corresponding figures** |
| --- | --- | --- | --- |
| The length of internode | 17 | 17 | Supplementary Fig. S2 |
| Rind penetrometer strength before tasseling | 20 | 20 | Fig.1C |
| Rind penetrometer strength one week after silking | 16 | 16 | Supplementary Fig. S3 |
| Vascular bundle characteristics | 5 | 5 | Fig.2 |
| Stalk cell wall components | 3 replicates with each contained 10 samples | 3 replicates with each contained 10 samples | Fig.3 |
| Metabolite detection | 3 replicates with each contained 3 samples | 2 replicates with each contained 3 samples | Fig.4 |
| RNA sequencing | Same samples with metabolite detection | Same samples with metabolite detection | Fig.5 |
| qRT-PCR | Same samples with metabolite detection | Same samples with metabolite detection | Supplementary Fig. S9 |
